# Targeting MicroRNA-21 in Chronic Kidney Disease: Lessons from the Lademirsen Story

**DOI:** 10.3390/medsci14050547

**Published:** 2026-09-07

**Authors:** Verica Stankovic Popovic, Aleksandar Sic, Selena Gajic, Dusan Vicentijevic, Ana Bontic, Jelena Pavlovic, Aleksandra Kezic, Marko Baralic

**Affiliations:** 1 Nephrology Clinic, University Clinical Center of Serbia, 11000 Belgrade, Serbia; vericasp@gmail.com (V.S.P.); selenagajic@yahoo.com (S.G.); bontic@gmail.com (A.B.); jelena@pavlovic.rs (J.P.); aleksandrakezic@yahoo.com (A.K.); 2 Faculty of Medicine, University of Belgrade, 11000 Belgrade, Serbia

**Keywords:** chronic kidney disease, microRNA-21, lademirsen, Alport syndrome, RNA therapeutics, kidney fibrosis, translational medicine, biomarker, antisense oligonucleotides

## Abstract

MicroRNA-21 (miR-21) has long been regarded as one of the most promising molecular targets in chronic kidney disease (CKD) because of its consistent upregulation across diverse renal disorders and its involvement in fibrosis, inflammation, oxidative stress, and metabolic dysfunction. Strong preclinical evidence demonstrated that inhibition of miR-21 reduced kidney injury, preserved renal function, and improved survival in multiple experimental models, particularly Alport syndrome, leading to the clinical development of the antisense oligonucleotide lademirsen. However, despite a compelling biological rationale and encouraging animal data, the phase 2 HERA trial failed to demonstrate a clinically meaningful effect on the rate of kidney function decline, resulting in discontinuation of the program. This review examines the biological functions of miR-21 in CKD, summarizes the experimental and clinical evidence that supported its therapeutic development, and critically analyzes the factors that may explain the discrepancy between preclinical success and clinical failure. Particular attention is given to the distinction between disease-associated biomarkers and true therapeutic drivers, the limitations of animal models, disease heterogeneity, timing of intervention, and the complex regulatory networks underlying progressive kidney fibrosis. Rather than representing the end of miR-21 research, the experience with lademirsen provides valuable insights into the challenges of translating RNA-based therapies into clinical practice. Future therapeutic strategies will likely require earlier intervention, improved patient selection, robust biomarkers of target engagement, and combination approaches that address the multifactorial nature of CKD. The lessons learned from the lademirsen program may help guide the development of the next generation of RNA therapeutics for kidney disease.

## 1. Introduction

Chronic kidney disease (CKD) is still one of the leading causes of morbidity and mortality worldwide, affecting hundreds of millions of individuals and placing a substantial burden on healthcare systems. Although the introduction of renin–angiotensin system inhibitors, sodium-glucose cotransporter-2 inhibitors and non-steroidal mineralocorticoid receptor antagonists has improved outcomes, many patients continue to experience progressive loss of kidney function. As a result, considerable effort has been directed toward identifying molecular pathways that could be targeted to slow or even stop CKD progression [1,2,3].

Among the emerging therapeutic strategies, microRNAs attracted particular attention because of their ability to regulate entire gene networks rather than individual signaling molecules. This characteristic appeared especially relevant in CKD, where fibrosis, inflammation, oxidative stress, metabolic dysfunction and maladaptive tissue repair interact through complex and interconnected pathways. Of the numerous microRNAs implicated in kidney disease, microRNA-21 (miR-21) rapidly emerged as one of the most promising candidates due to its consistent upregulation across diverse forms of renal injury [4].

Throughout the 2010s, experimental evidence suggested that miR-21 occupied a central position within the molecular machinery driving kidney fibrosis and progressive nephron loss. Inhibition of miR-21 improved renal histology, preserved kidney function, and prolonged survival in multiple animal models, particularly in Alport syndrome, where preclinical results were strikingly consistent. These findings generated considerable enthusiasm and positioned miR-21 as one of the first non-coding RNA targets to advance from experimental nephrology into clinical drug development [4,5].

The development of lademirsen, an antisense oligonucleotide designed to inhibit miR-21, was therefore viewed as a landmark step for RNA-based therapeutics in kidney disease. Expectations were high that targeting a molecule implicated in several pathogenic pathways simultaneously might overcome some of the limitations associated with conventional therapies. However, despite a strong biological rationale and encouraging preclinical data, the phase 2 HERA trial failed to demonstrate a meaningful benefit on kidney function decline in patients with Alport syndrome, leading to discontinuation of the program for futility. The failure was not primarily related to safety concerns, which makes the discrepancy between animal and human outcomes even more intriguing [5,6,7].

Rather than providing another narrative overview of miR-21 biology or the HERA trial, this review examines the complete lademirsen development program across successive stages of therapeutic target validation, including biological rationale, biomarker evidence, experimental validation, clinical testing, and post-trial interpretation. By critically evaluating the evidence supporting each stage, we identify where confidence in miR-21 as a therapeutic target was justified and where important translational uncertainties persisted despite encouraging preclinical findings. This stage-based analysis illustrates how apparently robust experimental evidence can fail to translate into clinical benefit and highlights broader considerations for therapeutic target validation and the development of future RNA-based therapies for chronic kidney disease. While previous reviews have primarily summarized the biological functions of miR-21 or the clinical experience with lademirsen, the present review critically evaluates therapeutic target validation across successive stages of translation and examines how cumulative translational uncertainties contributed to the failure of clinical efficacy.

## 2. Methodology

This narrative review was prepared through a comprehensive review of the published literature on miR-21, CKD and the clinical development of lademirsen. Relevant articles were identified primarily through searches of PubMed/MEDLINE, with additional references retrieved from the bibliographies of selected publications. Priority was given to experimental studies, clinical trials, systematic reviews and landmark papers addressing the biological role of miR-21, translational research, RNA-based therapeutics, and the HERA trial. The selected literature was critically evaluated and synthesized to provide an integrated overview of current evidence, with particular emphasis on the translational challenges that contributed to the discrepancy between preclinical success and clinical outcomes.

## 3. Biology of miR-21 in the Healthy and Diseased Kidney

MicroRNAs (miRNAs) are short non-coding RNA molecules, approximately 22 nucleotides in length, that regulate gene expression at the post-transcriptional level by binding to target messenger RNAs and promoting their degradation or translational repression [8]. Among the numerous miRNAs implicated in kidney biology, microRNA-21 (miR-21) has emerged as one of the most extensively studied due to its consistent upregulation across multiple forms of CKD and its involvement in pathways regulating fibrosis, inflammation, oxidative stress and cellular metabolism [8,9].

Under physiological conditions, miR-21 is expressed at relatively low levels in the healthy kidney and contributes to normal cellular homeostasis. Its expression can be detected in several renal cell populations, including tubular epithelial cells, podocytes, fibroblasts, endothelial cells and infiltrating immune cells. In response to kidney injury, however, miR-21 expression increases markedly, particularly within damaged tubular epithelial cells and activated interstitial fibroblasts. This upregulation has been seen in diabetic kidney disease, hypertensive nephropathy, glomerulonephritis, Alport syndrome and various experimental models of tubulointerstitial fibrosis, suggesting that miR-21 represents a common molecular response to chronic renal injury rather than a disease-specific phenomenon [8,10].

The biological significance of miR-21 lies in its ability to simultaneously regulate multiple signaling pathways involved in CKD progression. One of its best-characterized targets is SMAD7, an endogenous inhibitor of transforming growth factor-beta (TGF-β) signaling. By suppressing SMAD7 expression, miR-21 amplifies TGF-β/SMAD3 activity, resulting in enhanced extracellular matrix deposition, fibroblast activation and progressive renal fibrosis. Increased miR-21 expression is associated with enhanced collagen production and accelerated fibrotic remodeling of the kidney [11,12].

Another important target of miR-21 is a phosphatase and tensin homolog (PTEN), a negative regulator of the PI3K/AKT pathway. Suppression of PTEN by miR-21 promotes AKT activation, contributing to renal cell hypertrophy, extracellular matrix accumulation and fibrotic remodeling. In parallel, miR-21 directly targets SMAD7, an endogenous inhibitor of TGF-β signaling, thereby amplifying profibrotic TGF-β/SMAD3 activity. PTEN and SMAD7 suppression create a molecular environment that favors progressive renal fibrosis and functional decline in diabetic nephropathy and other forms of CKD [13,14].

Beyond its effects on fibrotic signaling, miR-21 also influences cellular metabolism [15]. MiR-21 promotes kidney fibrosis through suppression of genes involved in fatty acid oxidation and mitochondrial function, particularly pathways regulated by PPAR-α (peroxisome proliferator-activated receptor alpha) [16]. Reduced fatty acid oxidation leads to ATP depletion, lipid accumulation, and dedifferentiation of tubular epithelial cells, rendering them more susceptible to injury and maladaptive repair processes that contribute to progressive kidney fibrosis [17]. These observations expanded the understanding of miR-21 beyond a regulator of fibrotic signaling and highlighted its role in the metabolic reprogramming that accompanies CKD progression.

MiR-21 additionally contributes to renal inflammation through interactions with multiple cytokine and immune signaling pathways [12]. Increased miR-21 expression has been associated with inflammatory cell infiltration and sustained pro-inflammatory signaling within the injured kidney [10]. MiR-21 inhibition reduces the expression of inflammatory mediators, including TNF-α and IL-1β, while attenuating renal injury, supporting a role for miR-21 in linking chronic inflammation to progressive kidney damage [18].

The biological effects of miR-21 appear to be highly context-dependent. While transient miR-21 activation may represent an adaptive response following acute injury, persistent overexpression during chronic disease promotes maladaptive repair, fibrosis, and progressive nephron loss. Current evidence suggests that miR-21 cannot be regarded as either a primary pathogenic driver or merely a passive downstream marker of kidney injury. Instead, it appears to act primarily as a disease amplifier, reinforcing profibrotic, inflammatory, and metabolic pathways that sustain disease progression after injury has been initiated [19,20]. Its consistent upregulation across diverse kidney diseases, together with its central role in several interconnected pathogenic pathways, provided the biological rationale for therapeutic targeting. However, the HERA trial highlighted that biological relevance alone does not necessarily indicate that a molecule is indispensable for disease progression or that its inhibition will translate into clinical benefit [21].

## 4. Linking miR-21 to CKD Progression

### 4.1. Experimental Evidence

The rationale for targeting miR-21 originated from observations that its expression increased across virtually all major experimental models of CKD, regardless of the initiating injury. Although the initiating insults differed substantially, increased miR-21 expression was repeatedly observed in progressive kidney injury and was associated with worsening histological and functional outcomes. This reproducibility suggested that miR-21 might be a pathway involved in CKD progression rather than a disease-specific molecular abnormality [4,22].

A major turning point came from studies demonstrating that therapeutic inhibition of miR-21 could alter the natural history of experimental kidney disease. In murine models of Alport syndrome, anti-miR-21 treatment reduced albuminuria, attenuated glomerulosclerosis and tubulointerstitial fibrosis, preserved renal function, and significantly prolonged survival. Importantly, these benefits were observed despite the persistence of the underlying genetic defect, suggesting that miR-21 inhibition acted on downstream mechanisms driving disease progression [18].

The translational appeal of miR-21 was further strengthened by subsequent studies showing efficacy across different CKD phenotypes. Protective effects were reported in diabetic kidney disease, obstructive nephropathy, and other experimental models characterized by progressive fibrosis. Rather than influencing a single pathological process, miR-21 inhibition appeared to simultaneously improve several hallmarks of CKD progression, including structural injury, inflammatory activity, and functional decline. This broad spectrum of activity distinguished miR-21 from many previous therapeutic targets that were restricted to a single signaling pathway [23,24].

Another factor that fueled enthusiasm was the favorable preclinical safety profile. Long-term suppression of miR-21 in animal studies was generally well tolerated, with no major toxicity signals reported despite sustained inhibition. Combined with robust efficacy data, these findings provided a strong rationale for advancing anti-miR-21 therapies into human studies. By the late 2010s, miR-21 had become one of the most promising non-coding RNA targets in nephrology, ultimately leading to the clinical development of lademirsen [18,25].

### 4.2. Human Evidence

The translational rationale for targeting miR-21 was not based solely on animal studies. Evidence from human kidney disease consistently demonstrated increased miR-21 expression across multiple CKD etiologies, including diabetic kidney disease, IgA nephropathy, hypertensive nephropathy, focal segmental glomerulosclerosis and Alport syndrome [26,27]. Elevated miR-21 levels have been detected in kidney tissue, urine, circulating plasma, and extracellular vesicles, suggesting that miR-21 activation is a common feature of chronic renal injury rather than a disease-specific phenomenon [28,29]. MiR-21 expression was particularly enriched in areas of active tubular injury and interstitial fibrosis. The degree of miR-21 upregulation frequently correlated with histological fibrosis, proteinuria, and declining kidney function, supporting the hypothesis that miR-21 participates in pathways associated with CKD progression [30]. These findings appeared highly consistent with observations from experimental models and strengthened confidence that miR-21 represented a clinically relevant therapeutic target.

However, an important limitation emerged from these observations. Most human studies were cross-sectional or observational and therefore unable to establish causality. Although miR-21 consistently tracked with disease severity, fibrosis, and kidney dysfunction, such associations could not determine whether miR-21 was actively driving disease progression or simply reflecting ongoing tissue injury. This uncertainty would later become central to the interpretation of the negative lademirsen trial results and remains one of the key unresolved questions in the field.

### 4.3. Biomarker Studies and Clinical Correlates

As enthusiasm surrounding miR-21 continued to grow, attention gradually shifted from its biological role toward its potential clinical utility. Unlike many candidate biomarkers that require invasive tissue sampling, miR-21 could be detected in multiple readily accessible biological compartments, including plasma, urine, and urinary extracellular vesicles. This raised the possibility that molecular processes occurring within the kidney might be monitored non-invasively, offering an attractive alternative to repeated kidney biopsies [31,32].

The emergence of extracellular vesicle research further strengthened interest in miR-21. Because these vesicles originate from renal cells and carry molecular cargo reflecting ongoing cellular activity, they were increasingly viewed as a form of “liquid biopsy” capable of capturing disease-related molecular changes. Within this framework, miR-21 became one of the most extensively investigated miRNAs in urinary vesicles, linking experimental observations with clinically accessible biomarkers [33].

However, the growing biomarker literature also exposed an important paradox. The very characteristics that made miR-21 attractive, its sudden induction across diverse forms of kidney injury and its detectability in multiple biological fluids, also limited its specificity. MiR-21 is dysregulated in cardiovascular disease, malignancy, metabolic disorders, and numerous inflammatory conditions, raising concerns that elevated levels may reflect a generalized injury response rather than a kidney-specific pathogenic process. So, attention gradually shifted from the use of miR-21 as a standalone marker toward its incorporation into multimarker and extracellular-vesicle–based signatures [34].

This distinction proved particularly important in retrospect. While biomarker studies consistently demonstrated that miR-21 tracked disease activity, they could not establish whether the molecule occupied a causal position within CKD progression. The assumption that a strong biomarker necessarily represents an effective therapeutic target would later be challenged by the clinical experience with lademirsen, highlighting the fundamental difference between biological association and therapeutic vulnerability.

## 5. Therapeutic Targeting of miR-21: Preclinical Success

### 5.1. Anti-miR-21 Oligonucleotides

The consistent association of miR-21 with renal fibrosis and disease progression encouraged the development of therapies designed to inhibit its activity. Unlike conventional small-molecule drugs, anti-miR therapies are designed to bind mature microRNAs and prevent their interaction with downstream targets. Advances in oligonucleotide chemistry substantially improved stability and tissue exposure, allowing sustained inhibition of miR-21 after systemic administration and making therapeutic microRNA silencing a realistic clinical strategy [35,36].

Among the compounds developed for kidney disease, lademirsen (RG-012, later SAR339375) became the most advanced anti-miR-21 candidate. The drug was specifically designed to inhibit miR-21 activity within renal tissue and was initially developed for Alport syndrome, a rare genetic kidney disease characterized by progressive fibrosis and loss of kidney function. Because miR-21 expression is markedly increased in both human Alport syndrome and experimental disease models, the condition was considered an attractive setting for proof-of-concept evaluation of microRNA-targeted therapy [37,38].

Early preclinical studies demonstrated effective suppression of renal miR-21 activity together with restoration of metabolic pathways, attenuation of fibrosis and preservation of kidney function [27]. All of these findings generated considerable enthusiasm and established anti-miR-21 therapy as one of the first RNA-based therapeutic approaches to reach clinical development in nephrology.

### 5.2. Effects in Experimental CKD

The enthusiasm surrounding anti-miR-21 therapy was driven not only by consistent target engagement but also by the breadth of biological effects observed across experimental models of CKD. Unlike interventions directed at a single signaling pathway, miR-21 inhibition appeared to influence several interconnected processes that collectively drive disease progression, including fibrosis, inflammation, oxidative stress, and metabolic dysfunction. This multimodal activity was particularly attractive in CKD, where progressive nephron loss results from the interaction of numerous pathogenic mechanisms rather than a single dominant pathway [5,39].

One of the most reproducible findings was attenuation of renal fibrosis. Across models of diabetic kidney disease, unilateral ureteral obstruction, and hereditary nephropathies, suppression of miR-21 reduced extracellular matrix accumulation, decreased collagen deposition and limited activation of myofibroblasts. These effects were largely attributed to restoration of antifibrotic pathways normally suppressed by miR-21, including PTEN, SMAD7, and regulators of cellular metabolism. Antifibrotic benefits were observed even when treatment was initiated after kidney injury had already been established, suggesting that miR-21 contributed to disease propagation rather than merely reflecting existing damage [40].

Anti-miR-21 therapy also demonstrated significant anti-inflammatory effects. Experimental studies showed reduced macrophage infiltration and lower expression of pro-inflammatory mediators such as TNF-α, IL-1β, and NF-κB–associated signaling molecules following miR-21 suppression. More recent work has further linked miR-21 activity to the SPRY1/ERK/NF-κB axis, providing a mechanistic explanation for how miR-21 may amplify inflammatory responses and thereby accelerate fibrotic remodeling. The observation that inhibition of a single microRNA could simultaneously attenuate both fibrosis and inflammation strengthened the perception of miR-21 as a central regulatory node in CKD progression [5,41]. Oxidative stress and mitochondrial dysfunction represented another important component of the preclinical story. MiR-21 overexpression was associated with impaired fatty acid oxidation, mitochondrial injury, and increased susceptibility of tubular epithelial cells to chronic damage. Inhibition of miR-21 restored metabolic pathways involved in energy homeostasis and reduced oxidative stress markers, supporting the concept that miR-21 contributes to metabolic reprogramming during chronic kidney injury [16].

The remarkable reproducibility of these results across diverse experimental settings created the impression that miR-21 inhibition could modify core mechanisms of CKD progression. However, this apparent consistency should be interpreted with caution. Although the experimental models differed in the initiating cause of kidney injury, many shared important methodological characteristics, including early treatment initiation, genetically homogeneous animals, controlled laboratory conditions, and reliance on histological or survival endpoints rather than long-term clinical outcomes. The consistent efficacy observed across these models may have reflected shared experimental design and disease biology rather than demonstrating that miR-21 represents a universally indispensable driver of CKD progression. This body of evidence ultimately laid the foundation for the selection of Alport syndrome as the first clinical testing ground for anti-miR-21 therapy. The principal preclinical studies differed in animal models, treatment regimens, timing of intervention, background therapy, and study endpoints. A study-by-study comparison of these investigations alongside the HERA clinical trial is presented in Table 1 to facilitate direct comparison of the experimental designs and the potential factors contributing to the translational gap.

### 5.3. The Alport Syndrome Story

Among the various forms of CKD in which miR-21 was found to be dysregulated, Alport syndrome gradually emerged as the most attractive setting for clinical translation. Unlike diabetic kidney disease or hypertensive nephropathy, Alport syndrome is caused by pathogenic variants in COL4A3, COL4A4 or COL4A5 and follows a relatively predictable disease course. This combination of a clearly defined molecular origin and progressive kidney function decline created a favorable environment for testing novel disease-modifying therapies. By the early 2020s, Alport syndrome had become a major focus of therapeutic development, attracting approaches ranging from endothelin receptor antagonists and SGLT2 inhibitors to gene-editing and RNA-based technologies [42,43,44].

The growing interest in RNA therapeutics further strengthened the rationale for selecting Alport syndrome as an initial clinical target. Advances in antisense oligonucleotides, exon-skipping strategies, and other RNA-based platforms demonstrated that inherited kidney diseases could potentially be modified at the transcript level, even when direct correction of the underlying mutation remained challenging. Within this rapidly evolving field, miR-21 inhibition represented a fundamentally different strategy. Rather than attempting to repair the causative genetic defect, it sought to interfere with downstream pathways responsible for disease progression. This distinction was particularly appealing because it offered a mutation-independent approach that could theoretically be applied across different genetic forms of Alport syndrome [36,45].

Despite recognized uncertainties, including the inability of observational human studies to establish causality and the limited specificity of miR-21 as a biomarker, clinical development was considered justified. At the time, these uncertainties were not viewed as sufficient to preclude clinical testing because the available evidence consistently suggested that miR-21 contributed to pathways involved in disease progression rather than representing an isolated epiphenomenon. Moreover, therapeutic inhibition produced reproducible benefits across multiple experimental models, a strong mechanistic rationale supported the intervention, effective disease-modifying therapies for Alport syndrome were lacking, and early clinical studies demonstrated an acceptable safety profile. Investigators considered that the potential therapeutic benefit outweighed the remaining translational uncertainties, while recognizing that only randomized clinical trials could ultimately determine whether miR-21 represented a true therapeutic target rather than a disease-associated biomarker [18,25].

At the time lademirsen entered clinical development, the broader therapeutic landscape of Alport syndrome was also undergoing rapid transformation. Multiple experimental programs were exploring transcript-targeted therapies, exon-skipping approaches, gene replacement strategies, and surrogate endpoints designed to accelerate clinical trials. Against this background, anti-miR-21 therapy was viewed not simply as another candidate drug, but as one of the first opportunities to determine whether RNA-targeted interventions could successfully alter the course of progressive kidney disease in humans. The HERA program therefore represented more than a study of lademirsen itself; it became one of the earliest clinical evaluations of a microRNA-targeted therapy in nephrology [36,38,43,44].

## 6. Clinical Translation: The Rise and Fall of Lademirsen

### 6.1. From Experimental Models to Human Trials

The development of lademirsen occurred during a period of growing optimism surrounding RNA-based therapeutics. Advances in antisense oligonucleotides, small interfering RNAs, and messenger RNA technologies demonstrated that disease processes could be modified at the transcript level, creating new opportunities for conditions in which conventional pharmacological approaches had shown limited success. Kidney disease rapidly became one of the fields attracting interest because progressive renal dysfunction is driven by complex molecular networks that are often difficult to influence through single-target interventions [46]. Among the various non-coding RNAs implicated in kidney disease, miR-21 appeared particularly attractive from a translational perspective. Unlike many candidate targets restricted to a single pathway, miR-21 occupied a central position within interconnected networks regulating fibrosis, inflammation, mitochondrial homeostasis, oxidative stress, and cellular metabolism. This raised the possibility that inhibition of a single microRNA could simultaneously influence multiple mechanisms contributing to CKD progression. Such a systems-based therapeutic strategy represented a marked departure from traditional drug development, which had largely focused on individual receptors, enzymes, or cytokines [46,47].

Earlier generations of RNA therapeutics were limited by rapid degradation, poor tissue penetration, and concerns regarding long-term safety. Over the last decade, chemical modifications substantially improved nuclease resistance, pharmacokinetic stability, and tissue exposure, transforming antisense-based therapies from experimental tools into clinically viable drug platforms. The success of RNA-targeted treatments in several rare genetic disorders strengthened confidence that similar approaches could be applied to chronic kidney diseases [48,49].

At the same time, the broader field of microRNA therapeutics was maturing. Several candidate compounds had entered clinical development across oncology, cardiovascular medicine, infectious diseases, and inherited disorders. Although clinical progress remained slower than initially anticipated, these programs demonstrated that sustained modulation of disease-associated microRNAs was feasible in humans. They also highlighted challenges that would later become highly relevant in nephrology, including target selection, tissue-specific delivery, compensatory biological pathways, and the distinction between disease drivers and disease-associated biomarkers [50,51].

Against this background, lademirsen emerged as one of the most advanced microRNA-directed therapies in kidney disease. Its development reflected a broader belief that targeting master regulators of cellular injury might provide greater therapeutic benefit than inhibiting individual downstream pathways. By the time the HERA study was initiated, anti-miR-21 therapy had become more than a potential treatment for Alport syndrome. It represented one of the earliest opportunities to evaluate whether a microRNA-targeted therapy could modify the course of progressive kidney disease in humans [25,27,46].

### 6.2. The HERA Clinical Program

By the time lademirsen entered clinical development, Alport syndrome had become one of the few inherited kidney diseases with sufficiently established standards of care, well-defined genetic diagnosis, and predictable patterns of disease progression to support interventional trials. Recent international recommendations also emphasized the rapid evolution of the therapeutic landscape, with increasing interest in disease-modifying strategies capable of delaying kidney failure beyond conventional renin–angiotensin system blockade. Alport syndrome provided a unique opportunity to evaluate whether targeting a shared downstream pathway could complement, rather than replace, mutation-specific approaches [52].

Lademirsen was designed to interrupt the maladaptive molecular response that develops after the primary genetic defect has already initiated chronic injury. This represented an important conceptual shift. The underlying assumption was that progressive fibrosis, inflammation, and metabolic dysfunction might eventually become partially independent of the initiating mutation, allowing a single anti-miR-21 therapy to benefit patients across different genetic forms of Alport syndrome. Such a mutation-independent strategy distinguished lademirsen from many other investigational therapies under development at the time [18,27].

The HERA trial was consequently designed not simply to evaluate a new drug, but to test whether modulation of a regulatory microRNA could produce clinically meaningful benefit in human kidney disease. Adults with genetically confirmed Alport syndrome receiving optimized background therapy were randomized in a 2:1 ratio to weekly subcutaneous lademirsen (110 mg) or placebo in a double-blind, placebo-controlled phase 2 trial. The study enrolled 43 participants, included a 48-week double-blind treatment period followed by a 48-week open-label extension, and evaluated safety together with the annualized rate of eGFR decline as co-primary endpoints. Unlike many early RNA-based clinical programs that primarily focused on pharmacokinetics or biomarker modulation, HERA adopted the annual rate of eGFR decline as its principal efficacy endpoint, reflecting the expectation that successful target inhibition should ultimately translate into preservation of kidney function rather than molecular target engagement alone [25].

The study attracted considerable attention because it represented one of the earliest opportunities to determine whether sustained inhibition of a disease-associated microRNA could modify the natural history of a chronic fibrotic kidney disease. As later highlighted in expert commentaries, HERA provided important clinical experience with microRNA-targeted therapy in nephrology that extended beyond the evaluation of lademirsen itself. Regardless of its outcome, the study generated valuable insights into the challenges of translating RNA-based therapies from experimental models to clinical practice.

### 6.3. Clinical Outcomes and Trial Termination

The publication of the HERA trial results in 2024 marked an important turning point for RNA therapeutics in nephrology. After years of encouraging experimental data, lademirsen became one of the first microRNA-directed therapies to undergo randomized evaluation using kidney function decline as a clinically meaningful endpoint. Expectations were high because preclinical studies had consistently demonstrated reductions in fibrosis, preservation of renal architecture, and prolonged survival across several models of Alport syndrome [7,25]. The trial did not meet either of its co-primary objectives. Lademirsen failed to slow the annualized rate of eGFR decline compared with placebo, while no clinically meaningful differences were observed in secondary efficacy outcomes.

All clinical findings were unequivocally negative. Lademirsen was generally well tolerated, with an acceptable overall safety profile and no unexpected treatment-related toxicities. Most adverse events were mild to moderate and reflected the underlying disease population rather than a specific safety signal associated with anti-miR-21 therapy. However, favorable tolerability was not accompanied by clinical efficacy. The annual rate of eGFR decline was nearly identical in the lademirsen and placebo groups, and no significant differences were observed in kidney function at any study visit or in the proportion of patients reaching predefined thresholds of renal function loss. Also, treatment did not produce convincing improvements in secondary efficacy measures that would suggest delayed disease progression [25]. An analysis performed according to the predefined study protocol demonstrated a very low probability that continued enrollment or longer follow-up would alter the primary outcome. On the basis of these findings, the independent data monitoring process concluded that the study met futility criteria, and clinical development of lademirsen for Alport syndrome was discontinued before progression to a phase 3 trial. Importantly, the decision reflected lack of therapeutic benefit rather than unacceptable toxicity, distinguishing HERA from many previous drug development failures in nephrology [25].

The implications of HERA extended well beyond a single investigational therapy. Because lademirsen represented one of the most advanced microRNA-based drug programs in kidney disease, the negative outcome prompted broader reconsideration of how preclinical discoveries should be translated into human trials. Rather than being interpreted simply as the failure of one compound, the HERA trial has been viewed as an opportunity to identify potential limitations of current translational strategies and to generate hypotheses that may inform future RNA-based therapeutic development. Subsequent editorials emphasized that the greatest contribution of the trial may lie not in demonstrating efficacy, but in clarifying the biological and clinical questions that must be addressed before similar therapies can be successfully developed [7,53].

### 6.4. Why Did Translation Fail?

The negative outcome of the HERA trial raised a fundamental question that extends far beyond lademirsen itself. The biological rationale appeared convincing, target engagement was supported by extensive experimental work, and the therapy demonstrated an acceptable safety profile. Yet none of these strengths translated into measurable preservation of kidney function. The available evidence suggests that several biological and translational factors likely converged to limit clinical efficacy [7,25].

One important consideration is the distinction between disease association and disease causality. MiR-21 is consistently upregulated in virtually every major form of chronic kidney injury and correlates closely with fibrosis, proteinuria, and declining kidney function. However, molecules that reliably reflect disease severity are not necessarily indispensable drivers of disease progression. Persistent miR-21 expression may therefore represent an adaptive or secondary response to ongoing tissue injury rather than a molecular dependency that remains therapeutically reversible in established CKD [15,26,28].

Another explanation relates to the biological complexity of progressive renal fibrosis. Experimental studies often identified miR-21 as a key regulatory hub because its inhibition simultaneously influenced multiple downstream pathways, including TGF-β signaling, inflammation, mitochondrial metabolism, and oxidative stress. This broad biological activity initially suggested that targeting miR-21 might modify several core mechanisms of CKD progression simultaneously. However, regulation of multiple pathways does not necessarily mean that miR-21 functions as an indispensable upstream driver of disease. Rather, it appears to operate as one component of a highly interconnected profibrotic network in which several parallel signaling pathways can compensate when a single regulator is inhibited. In patients with advanced chronic kidney disease, these networks become increasingly redundant, allowing disease progression to continue despite successful modulation of one molecular target. Recent reviews of kidney fibrosis have increasingly highlighted this network-level redundancy as a major obstacle for precision therapies targeting single molecular regulators [5,16,24].

The timing of intervention may also have influenced therapeutic efficacy. Most preclinical studies initiated anti-miR-21 treatment relatively early, when fibrosis remained partially reversible and substantial nephron mass was preserved. By contrast, participants enrolled in HERA already demonstrated progressive kidney dysfunction despite optimized standard therapy, suggesting that irreversible structural injury had already developed. Once extensive extracellular matrix remodeling and nephron loss become established, modulation of a single regulatory microRNA may be insufficient to restore renal function, even if target inhibition is biologically successful. This concept is increasingly recognized across CKD drug development, where therapeutic windows may be substantially narrower than initially anticipated [7,25].

Several methodological differences between the preclinical studies and the HERA trial further complicate direct translation of experimental findings into clinical benefit. Most animal studies used genetically homogeneous models, initiated treatment during the early stages of disease, and evaluated histological changes, fibrosis, or survival under tightly controlled experimental conditions. In contrast, HERA enrolled adults with established Alport syndrome receiving optimized background renin–angiotensin system blockade and evaluated annualized eGFR decline as its primary clinical endpoint. Differences in disease stage, treatment timing, background therapy, outcome measures, and biological heterogeneity may all have reduced the likelihood that the robust effects observed in experimental models would translate into measurable clinical benefit.

The HERA experience highlights challenges in translating promising findings from animal models into clinical benefit in Alport syndrome. Whether similar limitations apply across other CKD etiologies and RNA-based therapies remains to be established. Murine models of Alport syndrome are genetically homogeneous, develop disease over a compressed time course, and lack many of the biological and environmental modifiers present in patients. Human disease is considerably more heterogeneous, with variation in genetic background, disease stage, concomitant therapies, and adaptive molecular responses. Consequently, interventions capable of producing robust effects in experimental systems may generate only modest or clinically undetectable benefits in heterogeneous patient populations. The experience with lademirsen therefore reinforces the need for translational strategies that incorporate human molecular profiling, predictive biomarkers, and earlier patient stratification before large efficacy trials are undertaken [7,25,54].

### 6.5. Was miR-21 the Wrong Target or the Wrong Strategy?

The disappointing outcome of the HERA trial inevitably raised questions about the therapeutic relevance of miR-21. However, interpreting the negative results as evidence that miR-21 is an invalid therapeutic target would likely represent an oversimplification. The biological evidence accumulated over more than a decade consistently identifies miR-21 as an important regulator of fibrosis, inflammation, oxidative stress, and metabolic dysfunction across multiple experimental models of kidney disease. These observations remain valid despite the failure of a single clinical program [18,55].

A more plausible interpretation is that the therapeutic strategy, rather than the molecular target itself, may have been insufficient to overcome the complexity of established chronic kidney disease. Progressive renal fibrosis is increasingly understood as a self-sustaining network of interconnected signaling pathways rather than the consequence of a single dominant molecular driver. Within such biological networks, individual regulators may function as highly connected hubs without representing indispensable bottlenecks. Successful inhibition of one pathway may produce measurable molecular effects while having only limited influence on the overall trajectory of disease progression because parallel profibrotic circuits continue to maintain tissue injury. These challenges are not unique to miR-21. Other microRNAs linked to CKD, such as miR-223, also show different expression patterns in kidney tissue and blood, illustrating the complexity of microRNA biology. This further emphasizes that promising biological findings do not always translate into successful therapies [56]. This systems-level view has become increasingly prominent in recent discussions of kidney fibrosis and RNA therapeutics [46,55].

The HERA experience also emphasizes that target engagement should not be considered synonymous with clinical efficacy. Modern RNA therapeutics are capable of achieving sustained suppression of their intended molecular targets, yet effective modulation of gene expression does not necessarily translate into preservation of organ function. In chronic diseases characterized by irreversible structural remodeling, the relationship between molecular correction and clinical benefit is likely to be nonlinear. By the time extensive nephron loss and extracellular matrix remodeling have occurred, eliminating one pathogenic signal may simply be insufficient to reverse the biological momentum of disease. Similar translational challenges have been recognized across several RNA-based therapeutic programs beyond nephrology, highlighting that delivery, timing, tissue accessibility, and disease stage may be as important as target selection itself [35,57].

Rather than discouraging further development of microRNA-directed therapies, the HERA trial may instead redefine the conditions under which these interventions are most likely to succeed. Future studies may benefit from prioritizing earlier stages of disease, when fibrotic remodeling remains biologically modifiable, integrating molecular biomarkers to identify patients with active target pathways, and incorporating evidence of renal target engagement whenever feasible. Equally important, RNA-based therapies may ultimately prove most effective as components of combination treatment strategies alongside established disease-modifying agents, including renin–angiotensin system inhibitors, SGLT2 inhibitors, and endothelin receptor antagonists. Such an approach recognizes that CKD is driven by highly interconnected biological networks, making coordinated modulation of multiple pathogenic pathways more realistic than expecting durable benefit from inhibition of a single molecular regulator [35,58,59].

One important lesson from the lademirsen program is that miR-21 may not have been the wrong target, but that successful translation requires more than identifying a biologically relevant molecule. The HERA experience suggests that future RNA-based therapeutic development may benefit from integrating molecular biology with disease timing, patient selection, target validation, and systems-level understanding of chronic kidney disease. In this context, HERA should be viewed not as the endpoint of miR-21 research, but as a pivotal experiment that has refined the translational roadmap for the next generation of RNA-based therapies [60].

## 7. Reassessing the Role of miR-21 in CKD

The clinical failure of lademirsen has not diminished the biological importance of miR-21, but it has prompted a reassessment of how its role in CKD is interpreted. Early experimental studies largely portrayed miR-21 as a master regulator of renal fibrosis whose inhibition might substantially alter disease progression. More recent evidence, however, supports a more nuanced view. Rather than acting as an isolated pathogenic switch, miR-21 appears to function within a broader regulatory landscape composed of multiple interacting non-coding RNAs and signaling pathways that collectively govern inflammation, fibrosis, cellular metabolism, and tissue repair. Large-scale analyses of miRNA profiling studies have demonstrated that CKD is characterized by coordinated dysregulation of numerous microRNAs, emphasizing that disease progression reflects complex regulatory networks rather than dependence on a single molecular mediator [61,62]. This shift in perspective has important implications for translational research. The consistent upregulation of miR-21 across diverse CKD phenotypes confirms its biological relevance, yet biological relevance alone does not necessarily confer therapeutic dominance. Molecules occupying central positions within regulatory networks may contribute substantially to disease pathogenesis while remaining insufficient as standalone therapeutic targets because parallel pathways can compensate when a single node is inhibited. The HERA trial therefore challenges not the pathogenic significance of miR-21 itself, but the assumption that modulation of one regulatory microRNA is sufficient to alter the course of an established, multifactorial chronic disease.

At the same time, the clinical value of miR-21 may extend beyond direct therapeutic inhibition. Increasing evidence suggests that miRNAs achieve greater clinical utility when incorporated into multimarker signatures rather than evaluated individually. Meta-analyses indicate that combined miRNA panels consistently outperform single microRNAs for CKD detection and risk stratification, particularly when urinary biomarkers are integrated with conventional clinical parameters. Within this framework, miR-21 may remain an important component of future molecular profiling strategies even if it is not used as an isolated therapeutic target [63,64].

The field is increasingly moving beyond searching for individual “master molecules” toward understanding coordinated regulatory networks that vary across disease stages, renal cell populations, and clinical phenotypes. In this context, the legacy of HERA is not the abandonment of miR-21, but a more realistic appreciation of how RNA biology should be translated into clinical practice. Future advances will likely depend less on identifying additional dysregulated microRNAs and more on defining which regulatory networks remain therapeutically actionable in specific patient populations.

## 8. Conclusions

MicroRNA-21 remains one of the best-studied molecular regulators of chronic kidney disease, but the experience with lademirsen has changed how its therapeutic potential should be viewed. The HERA trial showed that a strong biological rationale and convincing preclinical results do not necessarily translate into clinical benefit. At the same time, it reinforced an important distinction between molecules that are associated with disease progression and those that remain effective therapeutic targets in established CKD. Rather than closing the chapter on miR-21, HERA has provided valuable insight into the challenges of developing RNA-based therapies for kidney disease. Future progress may depend on earlier intervention, better patient selection, reliable biomarkers, and treatment strategies that account for the complexity of CKD rather than focusing on a single molecular pathway. The story of miR-21 may therefore be viewed not as one of failure, but of refinement. The experience gained from the HERA trial provides valuable insights that may inform the future development and evaluation of RNA-based therapies in CKD and other chronic diseases.

## Figures and Tables

**Table 1:** Comparison of key preclinical anti-miR-21 studies and the HERA clinical trial, highlighting experimental design and potential factors contributing to translational discrepancies.

Study	Disease Model	Anti-miR-21 Regimen	Treatment Timing	Background Therapy	Primary Endpoints	Main Findings	Potential Factors Influencing Translation
Gomez et al., 2015 [18]	Col4a3−/− Alport mice (129X1/SvJ)	Chemically modified anti-miR-21 ASO; SC; 12.5–50 mg/kg weekly or 25 mg/kg twice weekly	Treatment initiated at 3.5 weeks of age, before advanced fibrosis	None	BUN, albuminuria, survival, glomerulosclerosis, crescents, tubulointerstitial fibrosis, tubular injury, inflammation	Preserved renal function, reduced albuminuria, glomerulosclerosis, fibrosis, tubular injury and inflammation, restored metabolic pathways, and prolonged median survival (76 vs. 108 days).	Homogeneous genetic model; treatment initiated before advanced fibrosis; controlled experimental conditions; no background standard-of-care therapy
Kölling et al., 2017 [20]	Streptozotocin-induced diabetic nephropathy (SV129 mice)	LNA-modified anti-miR-21 (LNA-21); administered at weeks 2 and 5 after diabetes induction	Early intervention after confirmation of hyperglycemia but before advanced nephropathy	None	Mesangial expansion, interstitial fibrosis, macrophage infiltration, podocyte loss, albuminuria, renal fibrotic and inflammatory gene expression	Reduced mesangial expansion, tubulointerstitial fibrosis, macrophage infiltration, podocyte loss, and albuminuria. Restored Cdc25a/Cdk6 expression and reduced fibrotic and inflammatory gene expression.	Prevention-oriented design in an experimental diabetic model; treatment before advanced structural damage; no concomitant standard CKD therapy
Rubel et al., 2022 [27]	Two Col4a3−/− Alport mouse models (129/SvJ and F1 hybrid)	Lademirsen; 24.5 mg/kg SC twice weekly (129/SvJ) or 50 mg/kg SC weekly (F1 hybrid)	Early intervention before overt renal dysfunction	Ramipril alone or ramipril + lademirsen	Survival, renal function (BUN, proteinuria), fibrosis, inflammation, miR-21 expression, renal transcriptomics	Combination therapy produced additive nephroprotective effects beyond ACE inhibition alone, improving survival, renal function, proteinuria, fibrosis, inflammation, and renal transcriptomic profiles.	Early treatment in genetically homogeneous models; controlled environment; additive benefit demonstrated with ACE inhibition before advanced disease
Gale et al., 2024 (HERA) [25]	Adults with genetically confirmed Alport syndrome (Phase 2 randomized, double-blind, placebo-controlled trial)	Lademirsen 110 mg SC once weekly	Established CKD; 48-week double-blind treatment	Stable ACE inhibitor or ARB therapy in all participants	Annualized eGFR slope, safety; secondary renal outcomes	Lademirsen was well tolerated but did not significantly slow kidney function decline compared with placebo. The study was terminated early for futility.	Established human CKD with greater biological heterogeneity; optimized background therapy; clinically meaningful endpoint (eGFR slope); treatment initiated after disease establishment rather than during early pathogenesis.

Abbreviations: ACE, angiotensin-converting enzyme; ARB, angiotensin receptor blocker; ASO, antisense oligonucleotide; BUN, blood urea nitrogen; CKD, chronic kidney disease; eGFR, estimated glomerular filtration rate; LNA, locked nucleic acid; SC, subcutaneous.

## Data Availability

No new data were created or analyzed in this study. Data sharing is not applicable to this article.

## References

[1] Francis A., Harhay M.N., Ong A.C.M., Tummalapalli S.L., Ortiz A., Fogo A.B., Fliser D., Roy-Chaudhury P., Fontana M., Nangaku M. (2024). Chronic Kidney Disease and the Global Public Health Agenda: An International Consensus. Nat. Rev. Nephrol..

[2] Rastogi A., Collins A., Kelepouris E., Kotzker W., Middleton J.P., Rajpal M., Roy-Chaudhury P., Chertow G.M. (2024). Practical Considerations and Implementation of Sodium-Glucose Co-Transporter-2 Inhibitors in Chronic Kidney Disease: Who, When, and How? A Position Statement by Nephrologists. J. Prim. Care Community Health.

[3] (2024). Kidney Disease: Improving Global Outcomes (KDIGO) CKD Work Group. KDIGO 2024 Clinical Practice Guideline for the Evaluation and Management of Chronic Kidney Disease. Kidney Int..

[4] Rodzoń-Norwicz M., Kogut P., Sowa-Kućma M., Gala-Błądzińska A. (2025). What a Modern Physician Should Know About microRNAs in the Diagnosis and Treatment of Diabetic Kidney Disease. Int. J. Mol. Sci..

[5] Sakuma H., Kawaguchi S., Kobayashi Y., Koizumi A., Nakagawa N. (2026). MicroRNA Regulation in Kidney Interstitial Fibrosis. Epigenomes.

[6] Bravo-Vázquez L.A., Paul S., Colín-Jurado M.G., Márquez-Gallardo L.D., Castañón-Cortés L.G., Banerjee A., Pathak S., Duttaroy A.K. (2024). Exploring the Therapeutic Significance of microRNAs and lncRNAs in Kidney Diseases. Genes.

[7] Weinstock B.A. (2024). Lessons Learned from HERA: The First Alport Syndrome Therapeutic Clinical Trial. Clin. J. Am. Soc. Nephrol..

[8] Peters L.J.F., Floege J., Biessen E.A.L., Jankowski J., van der Vorst E.P.C. (2020). MicroRNAs in Chronic Kidney Disease: Four Candidates for Clinical Application. Int. J. Mol. Sci..

[9] Gluba-Sagr A., Franczyk B., Rysz-Górzyńska M., Ławiński J., Rysz J. (2023). The Role of miRNA in Renal Fibrosis Leading to Chronic Kidney Disease. Biomedicines.

[10] Liu S., Wu W., Liao J., Tang F., Gao G., Peng J., Fu X., Zhan Y., Chen Z., Xu W. (2022). MicroRNA-21: A Critical Pathogenic Factor of Diabetic Nephropathy. Front. Endocrinol..

[11] McClelland A.D., Herman-Edelstein M., Komers R., Jha J.C., Winbanks C.E., Hagiwara S., Gregorevic P., Kantharidis P., Cooper M.E. (2015). miR-21 Promotes Renal Fibrosis in Diabetic Nephropathy by Targeting PTEN and SMAD7. Clin. Sci..

[12] Loboda A., Sobczak M., Jozkowicz A., Dulak J. (2016). TGF-β1/Smads and miR-21 in Renal Fibrosis and Inflammation. Mediat. Inflamm..

[13] Dhas Y., Arshad N., Biswas N., Jones L.D., Ashili S. (2023). MicroRNA-21 Silencing in Diabetic Nephropathy: Insights on Therapeutic Strategies. Biomedicines.

[14] Zou Z., Zhou N., Zhang C. (2026). miRNA in the Progression of Diabetic Kidney Disease: New Insight. Int. J. Mol. Sci..

[15] Larrue R., Fellah S., Van der Hauwaert C., Hennino M.-F., Perrais M., Lionet A., Glowacki F., Pottier N., Cauffiez C. (2022). The Versatile Role of miR-21 in Renal Homeostasis and Diseases. Cells.

[16] Chau B.N., Xin C., Hartner J., Ren S., Castano A.P., Linn G., Li J., Tran P.T., Kaimal V., Huang X. (2012). MicroRNA-21 Promotes Fibrosis of the Kidney by Silencing Metabolic Pathways. Sci. Transl. Med..

[17] Kang H.M., Ahn S.H., Choi P., Ko Y.A., Han S.H., Chinga F., Park A.S., Tao J., Sharma K., Pullman J. (2015). Defective Fatty Acid Oxidation in Renal Tubular Epithelial Cells Has a Key Role in Kidney Fibrosis Development. Nat. Med..

[18] Gomez I.G., MacKenna D.A., Johnson B.G., Kaimal V., Roach A.M., Ren S., Nakagawa N., Xin C., Newitt R., Pandya S. (2015). Anti-microRNA-21 Oligonucleotides Prevent Alport Nephropathy Progression by Stimulating Metabolic Pathways. J. Clin. Investig..

[19] Kumarswamy R., Volkmann I., Thum T. (2011). Regulation and Function of miRNA-21 in Health and Disease. RNA Biol..

[20] Kölling M., Kaucsar T., Schauerte C., Hübner A., Dettling A., Park J.K., Busch M., Wulff X., Meier M., Scherf K. (2017). Therapeutic miR-21 Silencing Ameliorates Diabetic Kidney Disease in Mice. Mol. Ther..

[21] Patel V., Noureddine L. (2012). MicroRNAs and Fibrosis. Curr. Opin. Nephrol. Hypertens..

[22] Skourtsidis K., Ioannou D., Kiosis G., Stergiou K., Georgaki M.N., Papamitsou T., Karachrysafi S. (2026). Long Non-Coding RNAs and Micro RNAs in Chronic Kidney Disease: Recent Advances and Future Directions—A 5-Year Systematic Review. Life.

[23] Noda Y., Kato N., Sato F., Suzuki Y., Tsubota S., Kitai H., Komatsu S., Tanaka A., Sato Y., Maeda K. (2025). Ameliorative Effect of an Anti-MicroRNA-21 Oligonucleotide on Animal and Human Models of Cystic Kidney Disease. Kidney360.

[24] Guo Y., Feng Y., Wu H., Gao H. (2026). The Role of Non-Coding RNAs in the Pathogenesis and Progression of Diabetic Kidney Disease. Int. J. Mol. Sci..

[25] Gale D.P., Gross O., Wang F., de la Rosa R.J.E., Hall M., Sayer J.A., Appel G., Hariri A., Liu S., Maski M. (2024). A Randomized Controlled Clinical Trial Testing Effects of Lademirsen on Kidney Function Decline in Adults with Alport Syndrome. Clin. J. Am. Soc. Nephrol..

[26] Motshwari D.D., Matshazi D.M., Erasmus R.T., Kengne A.P., Matsha T.E., George C. (2023). MicroRNAs Associated with Chronic Kidney Disease in the General Population and High-Risk Subgroups—A Systematic Review. Int. J. Mol. Sci..

[27] Rubel D., Boulanger J., Craciun F., Xu E.Y., Zhang Y., Phillips L., Callahan M., Weber W., Song W., Ngai N. (2022). Anti-microRNA-21 Therapy on Top of ACE Inhibition Delays Renal Failure in Alport Syndrome Mouse Models. Cells.

[28] Zang J., Maxwell A.P., Simpson D.A., McKay G.J. (2019). Differential Expression of Urinary Exosomal MicroRNAs miR-21-5p and miR-30b-5p in Individuals with Diabetic Kidney Disease. Sci. Rep..

[29] Li Q., Zhang Z., Yin M., Cui C., Zhang Y., Wang Y., Liu F. (2022). What Do We Actually Know about Exosomal microRNAs in Kidney Diseases?. Front. Physiol..

[30] Hennino M.F., Buob D., Van der Hauwaert C., Gnemmi V., Jomaa Z., Pottier N., Savary G., Drumez E., Noël C., Cauffiez C. (2016). miR-21-5p Renal Expression Is Associated with Fibrosis and Renal Survival in Patients with IgA Nephropathy. Sci. Rep..

[31] Delrue C., De Bruyne S., Speeckaert R., Speeckaert M.M. (2023). Urinary Extracellular Vesicles in Chronic Kidney Disease: From Bench to Bedside?. Diagnostics.

[32] Schnobrich L., Castrop H. (2025). Urinary Microvesicles: A Window into the Kidney. Clin. Kidney J..

[33] Zapała B., Kamińska A., Piwowar M., Paziewska A., Gala-Błądzińska A., Stępień E.Ł. (2023). miRNA Signature of Urine Extracellular Vesicles Shows the Involvement of Inflammatory and Apoptotic Processes in Diabetic Chronic Kidney Disease. Pharm. Res..

[34] Ata A., Ezzat D., Sherkawy H.S., Ahmed A.E., Afify M., Abdelmaksoud M.D., Azazy S., Gouda W. (2025). Impact of Micro-RNAs as biomarkers for end-stage renal disease related to hypertension and diabetes. Sci. Rep..

[35] Huang C.-K., Bär C., Thum T. (2020). miR-21, Mediator, and Potential Therapeutic Target in the Cardiorenal Syndrome. Front. Pharmacol..

[36] Schena F.P., Pasculli E. (2025). RNA Therapeutics in Kidney Diseases: Prospects and Current Status. Clin. Kidney J..

[37] U.S. National Library of Medicine A Study of Lademirsen (SAR339375) in Alport Syndrome (HERA); ClinicalTrials.gov Identifier: NCT03373786. NCT03373786.

[38] Shevelev A., Pozdniakova N., Generalov E., Tarasova O. (2025). siRNA Therapeutics for the Treatment of Hereditary Diseases and Other Conditions: A Review. Int. J. Mol. Sci..

[39] Abbad L., Esteve E., Chatziantoniou C. (2025). Advances and Challenges in Kidney Fibrosis Therapeutics. Nat. Rev. Nephrol..

[40] Zhong X., Chung A.C.K., Chen H.-Y., Meng X.-M., Lan H.Y. (2011). Smad3-Mediated Upregulation of miR-21 Promotes Renal Fibrosis. J. Am. Soc. Nephrol..

[41] Liu E., Sun X., Liu Q., Jin D., Li G., Zhao H., Sun H., Du Y. (2026). Erythropoietin Alleviates Obstructive Renal Fibrosis by Regulating Immunity and Inflammation through miR-21-5p/SPRY1/ERK1/2/NF-κB Pathway Inhibition. Front. Mol. Biosci..

[42] Chavez E., Rodriguez J., Drexler Y., Fornoni A. (2022). Novel Therapies for Alport Syndrome. Front. Med..

[43] Lo Re C., Kim J.J., Fornoni A. (2026). From RAAS Blockade to Regenerative Medicine: Evolving Treatment Strategies in Alport Syndrome. Pediatr. Nephrol..

[44] Oates T.M., Barua M., Gear S., Turner A.N., Lennon R., Kai H., Gale D.P., Aksenova M., Savige J., Gross O. (2026). Update on Alport Syndrome: The Report of the 2024 International Workshop on Alport Syndrome. Kidney Int. Rep..

[45] Yamamura T., Horinouchi T., Adachi T., Terakawa M., Takaoka Y., Omachi K., Takasato M., Takaishi K., Shoji T., Onishi Y. (2020). Development of an Exon Skipping Therapy for X-Linked Alport Syndrome with Truncating Variants in *COL4A5*. Nat. Commun..

[46] Hu L., Jin T., Zhang N., Ding J., Li L. (2025). RNA-Based Therapies in Kidney Diseases. J. Inflamm. Res..

[47] Lv W., Fan F., Wang Y., Gonzalez-Fernandez E., Wang C., Yang L., Booz G.W., Roman R.J. (2018). Therapeutic Potential of microRNAs for the Treatment of Renal Fibrosis and CKD. Physiol. Genomics.

[48] Kim H., Kim S., Lee D., Lee D., Yoon J., Lee H. (2024). Oligonucleotide therapeutics and their chemical modification strategies for clinical applications. J. Pharm. Investig..

[49] Shen X., Corey D.R. (2018). Chemistry, mechanism and clinical status of antisense oligonucleotides and duplex RNAs. Nucleic Acids Res..

[50] Ye C., Zheng J. (2025). Cancer therapeutics hope: microRNA clinical practice and perspectives. Vis. Cancer Med..

[51] Makkar S.K. (2025). Advances in RNA-based therapeutics: Current breakthroughs, clinical translation, and future perspectives. Front. Genet..

[52] Torra R., Lipska-Zietkiewicz B., Acke F., Antignac C., Becker J.U., Cornec-Le Gall E., van Eerde A.M., Feltgen N., Ferrari R., Gale D.P. (2025). Diagnosis, management and treatment of the Alport syndrome–2024 guideline on behalf of ERKNet, ERA and ESPN. Nephrol. Dial. Transplant..

[53] Alport Syndrome Foundation (2024). Research Summary.

[54] Kanbay M., Copur S., Capasso G., Turkmen K., Delanaye P., Bruchfeld A., Abd ElHafeez S., Ferro C.J., Verhaar M.C., Pesce F. (2026). The evolving field of nephrology: What comes next? A report from the European Renal Association Scientific Advisory Board. Clin. Kidney J..

[55] Todorović P., Pavlović N., Maglica M., Bajt P., Kelam N., Raguž F., Vukojević K. (2025). Non-Coding RNAs as Emerging Regulators in Kidney Pathophysiology: From Molecular Mechanisms to Therapeutic Potential. Genes.

[56] Prins J., Biscans A., van Zonneveld A.J., Frazier K.S., van der Veer E.P. (2026). RNA-Based Therapeutic Opportunities for the Treatment of Kidney Diseases. Nat. Rev. Nephrol..

[57] Gysi D.M., Barabási A.-L. (2022). Non-Coding RNAs Improve the Predictive Power of Network Medicine. arXiv.

[58] Li M., Xie C., Qiu Y., Li X., Han G., Ma N., Wang G. (2026). Progress in mechanistic and clinical translational research of endothelin A receptor antagonists in the treatment of diabetic kidney disease: A narrative review. Front. Endocrinol..

[59] Moldakhmetova S., Bimurat B., Berdaly A., Makhammajanov Z., Salybekov A.A., Bukasov R., Gaipov A. (2026). Emerging Urinary Biomarkers and Innovative Technologies for the Early Detection and Personalized Management of Chronic Kidney Disease. Int. J. Mol. Sci..

[60] Shahid U. (2025). Advances in RNA therapeutics: Classes, innovations and clinical applications. J. Precis. Med. Health Dis..

[61] Garmaa G., Bunduc S., Kói T., Hegyi P., Csupor D., Ganbat D., Dembrovszky F., Meznerics F.A., Nasirzadeh A., Barbagallo C. (2024). A Systematic Review and Meta-Analysis of microRNA Profiling Studies in Chronic Kidney Diseases. Non-Coding RNA.

[62] He K., Zhou X., Zhao J., Du H., Guo J., Deng R., Wang J. (2024). Identification and Functional Mechanism Verification of Novel MicroRNAs Associated with the Fibrosis Progression in Chronic Kidney Disease. Biochem. Genet..

[63] Jiang L., Li L., Zhang K., Zheng L., Zhang X., Hou Y., Cao M., Wang Y. (2025). A systematic review and meta-analysis of microRNAs in the diagnosis of early diabetic kidney disease. Front. Endocrinol..

[64] Garmaa G., Nagy R., Kói T., To U.N.D., Gergő D., Kleiner D., Csupor D., Hegyi P., Kökény G. (2024). Panel miRNAs are potential diagnostic markers for chronic kidney diseases: A systematic review and meta-analysis. BMC Nephrol..

